# Passivating Surface States on Water Splitting Cuprous Oxide Photocatalyst with Bismuth Decoration

**DOI:** 10.3390/molecules24224156

**Published:** 2019-11-16

**Authors:** Yuhong Huang, Hongkuan Yuan, Hong Chen

**Affiliations:** School of Physical Science and Technology, Southwest University, Chongqing 400715, China; huangyh211@163.com (Y.H.); yhk10@swu.edu.cn (H.Y.)

**Keywords:** cuprous oxide surface, adsorption, photocatalysis, hybrid density functional

## Abstract

To enhance the visible light photocatalystic activity of Cu_2_O(100) surface, we performed first-principles calculations on the structural, electronic and optical properties of a bismuth (Bi)-decorated Cu_2_O(100) surface (Bi@Cu_2_O(100)). It is shown that the Bi prefer to be loaded to the hollow sites among four surface oxygen atoms and tend to individual dispersion instead of aggregating on the surface due to the lowest formation energy and larger distance between two Bi atoms at the surface than the Bi clusters; the coverage of around 0.25 monolayer Bi atoms can effectively eliminate the surface states and modify the band edges to satisfy the angular momentum selection rules for light excited transition of electrons, and the loaded Bi atoms contribute to the separation of photogenerated electron-holes. The relative positions between the band edges and the redox potentials are suitable for photocatalytic hydrogen production from the redox water, and moreover, the optical absorption spectrum indicates a positive response of the Bi_0.25_@Cu_2_O(100) to visible light, implying that the Bi_0.25_@Cu_2_O(100) is a promising visible light photocatalyst.

## 1. Introduction

Cuprous oxide (Cu_2_O) is a promising candidate as a native p-type oxide semiconductor with a direct band gap of 2.17 eV in the field of gas sensors [1,2,3], solar energy conversion [4,5,6,7] and photocatalysis [8,9,10,11]. Cu_2_O is characterized by low toxicity, environmental acceptability, and low-cost elemental compositions because they are very abundant in the crust, which makes it promising for achieving large-scale industrial production to solve environmental and energy problems [10,12,13,14]. The photocatalytic technology based on semiconductor photocatalyst is considered as an ideal way to solve the energy and environmental problems fundamentally by using solar energy [15,16]. The ideal photocatalytic materials should be able to make full use of visible light for electrochemical splitting of water and decomposition of organic pollutants [17,18,19]. As a semiconductor photocatalyst, Cu_2_O has the advantages of absorption of visible light, proper edge position and good optical stability [20,21]. Visible light accounts for the majority of sunlight, which suggest that Cu_2_O can make full use of solar energy. However, the even-parity symmetry of the valence band maximum and conduction band minimum states in Cu_2_O prohibits the band edge radiative transition, which has hindered its potential use in optical applications [10,22,23].

Many Cu_2_O architectures with various morphologies have been successfully synthesized with the development of nanomaterials science and nanotechnology [24,25,26,27,28]. Perspective and progress on polyhedral Cu_2_O nanostructures also have received extensive attention [25,29,30]. Moreover, it was reported by Huang et al. [31] that the stability of Cu_2_O crystal planes follows the order of {100}≫{111}>{110} in weak acid solution (pH = 3.5). However, the development of Cu_2_O semiconductor photocatalyst has been an ongoing challenge because catalytic properties of nanocrystals are highly related to their exposed surfaces, which is the main place for the photocatalytic reaction. For the experimentally facet-dependent photocatalytic activity of Cu_2_O, we give an explanation by calculating the electronic structure of the Cu_2_O low-index surface. Especially, the surface states of the Cu_2_O(100) facet hinders its photocatalytic activity. Passivating surface states has been successful in modifying the surface of hematite [32,33,34], as far as we know, there is no report about the surface state passivation of Cu_2_O(100).

In this paper, we propose a strategy to modify the electronic structure of Cu_2_O(100) surface with depositing Bi atoms to investigate whether the surface decoration can improve the photocatalytic performance of Cu_2_O(100) surface, after analyzing the electronic structures of the Cu_2_O(100) surface. The electronic and optical properties of the Bi-loaded Cu_2_O(100) surface is investigated by using hybrid density functional theory. In addition, we also discuss the adsorption structure, adsorption energy, band edge potential and optical properties.

## 2. Computational Detail

**Figure 1:**
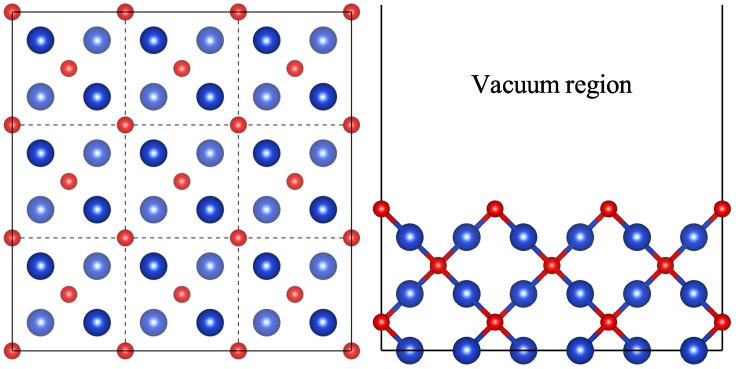
The 3 × 3 × 1 supercell structure for clean Cu_2_O(100) surface. The red and blue spheres represent oxygen atoms and copper atoms, respectively.

The Cu_2_O(100) surface is modeled by periodic six-layer slab models, and each slab is separated by a 15 Å vacuum layer to minimize interactions between the slabs. The surface structures are modeled with p (2 × 2) and p (3 × 3) supercells (Figure 1) to simulate different surface coverages. All periodic slab calculations based on density functional theory are performed using the Vinenna ab initio simulation package (VASP) [35,36]. The exchange and correlation interactions are modeled using the Perdew-Burke-Ernzerhof functional [37] within the generalized gradient approximation (GGA) in geometry optimization and total energy calculations [38]. The projector-augmented-wave (PAW) method is used for the description of electron-ion interaction. The wave functions of the valence electrons are expanded using a plane-wave basis set within a specified kinetic cutoff energy of 400 eV. The surface Brillouin Zone integrations are performed using 3 × 3 × 1 Monkhorst-Pack [39] k-points, the energy convergence criterion of 10^−5^ eV, and the force convergence thresholds of 0.02 eV/Å have been found to be sufficient for structural relaxation. To accurately describe the electronic structures and optical properties, the hybrid density functional as prescribed by Heyd-Scuseria-Ernzerhof (HSE06) [40,41] has been used in the present work. During the geometry optimization, the three topmost layers are allowed to fully relax, whereas the bottom three layers are fixed. To compensate the dipole effects along the z-direction, a dipole vector with the same value in the opposite direction is introduced.

## 3. Results and Discussion

### 3.1. Surface Property

**Figure 2:**
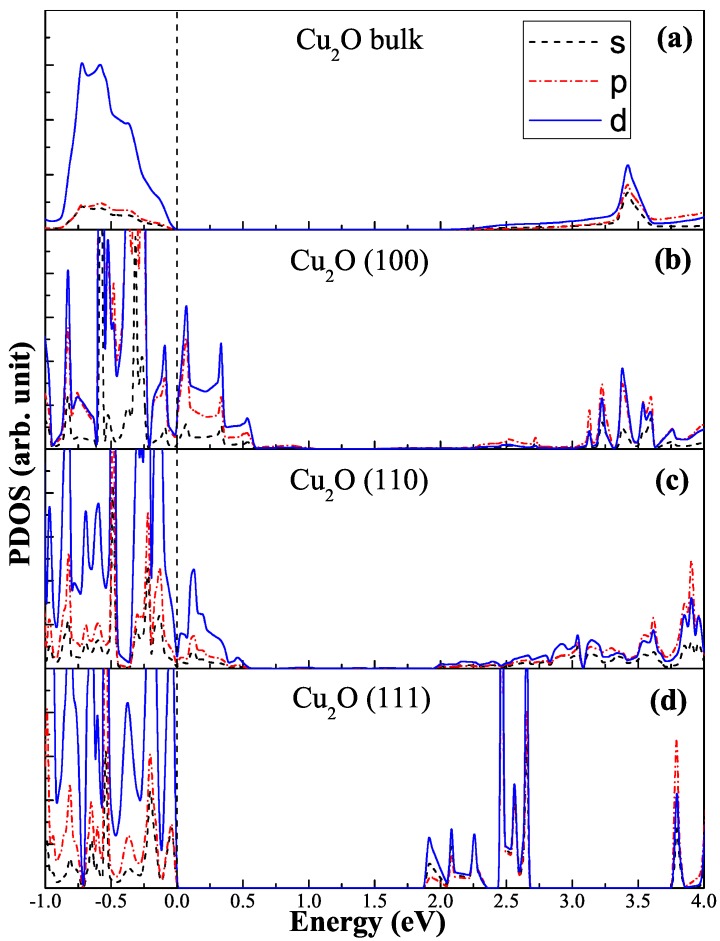
The projected density of states (PDOS) for Cu_2_O low-index surfaces.

In order to investigate the photocatalytic activity, we first calculate the electronic structures of the low-index surface of Cu_2_O. Figure 2 presents the projected density of states (PDOS) for Cu_2_O blocks and three low-index surfaces. They have a band gap of about 2 eV, which is good for meeting the photocatalytic requirements of visible light. One noteworthy thing is their band edge component, which is rarely considered a factor that affects the photocatalytic activity. As shown in Figure 2a, the band edges of Cu_2_O bulk are mainly composed of *d*-orbitals. Because the limitation of the dipole transition, only a small amount of electrons can transit to the *p*-orbitals of the conduction bandedge under light irradiation, which become the main factor to suppress the photocatalytic activity. In the bandgap of the (100) and (110) surfaces in Figure 2b,c, there are some empty bands which are not occupied by electrons, due to the reduction of the coordination number of the surface atoms. These empty bands can induce recombination of carriers to further reduce the quantum yield. For the (111) surface in Figure 2d, there are no empty states in the bandgap, but still face similar dipole transition problems.

### 3.2. Adsorption Energy

**Figure 3:**
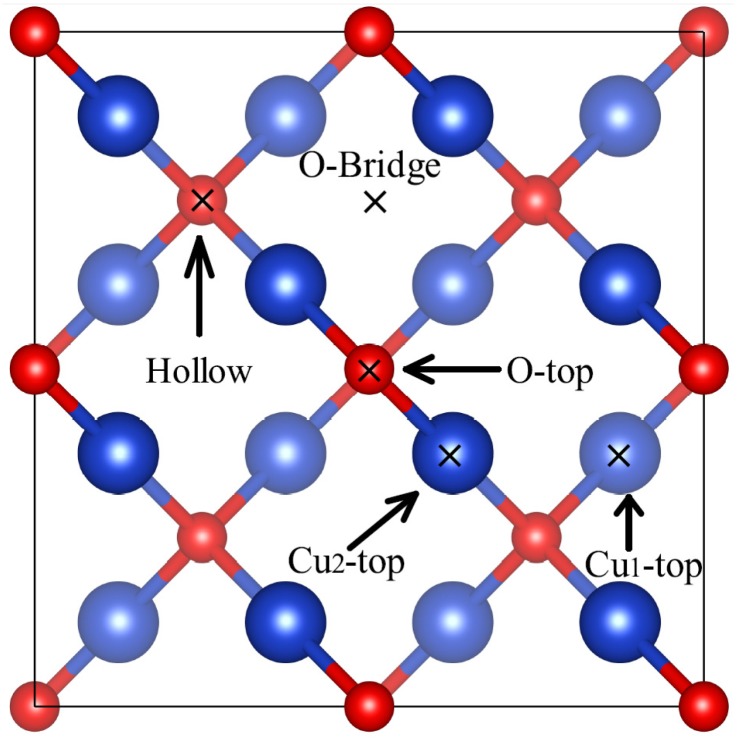
Five different adsorption sites on the Cu_2_O(100) surface, including O-top, O-bridge, hollow, Cu_1_-top, Cu_2_-top. The red and blue spheres represent oxygen atoms and copper atoms, respectively.

The adsorption structures of the Bi on different symmetry sites of the Cu_2_O(100) surface are investigated. In our calculations, five different adsorption sites on the Cu_2_O(100) surface are modeled: the top site of the surface oxygen atoms (O-top), the bridge site of the surface oxygen atoms (O-bridge), the hollow site among four surface oxygen atoms (hollow), the top site of the subsurface copper (Cu_1_-top) atoms and the top site of the fourth layer copper (Cu_2_-top) atoms (Figure 3). We now examine the energetic and thermodynamical stability of the Bi loaded Cu_2_O(100) surface (Bi@Cu_2_O(100)) by calculating the cohesive energy per unit area,

(1) $$E_{c}=\left[E_{slab+Bi}-n_{Cu}E_{Cu}-n_{O}E_{O}-n_{Bi}E_{Bi}\right]/S,$$

and the adsorption energy pere unit area [42],

(2) $$E_{av}=\left[E_{slab+Bi}-E_{slab}-n_{Bi}E_{Bi}\right)]/S.$$

Here, $n_{Cu/O/Bi}$ is the total number of Cu/O/Bi atoms in the decorated system, $E_{Cu/O/Bi}$ is the total energy of the isolated Cu/O/Bi atom, *S* is the area of the surface, $E_{slab+Bi}$ and $E_{slab}$ are the total energies of the Cu_2_O(100) surface with Bi adatoms and the clean Cu_2_O(100) surface, respectively.

The calculated $E_{c}$ and $E_{av}$ are presented in Table 1 for the Bi loaded Cu_2_O(100) surface. From the second to sixth row of Table 1, one Bi atom is absorbed at five possible sites of the surface. The $E_{av}$ of these systems are all negative, which indicates that they are stable structures. The adsorption of Bi atom on the hollow site is the most stable due to the lowest adsorption energy of −0.08 eV. Such system is more stable than the pristine Cu_2_O(100), given that its $E_{c}$ is 0.08 eV lower than that of pristine Cu_2_O(100). Moreover, it can be seen from the last four rows of Table 1 that the absolute adsorption energy increases as the surface coverage increases from 0.11 to 0.33 ML, which reflects that the structural stability of the Bi loaded Cu_2_O(100) surface can be improved by increasing the surface coverage. In the last two rows of Table 1, the two and three Bi atoms are simultaneously adsorbed at the hollow sites on the surface oxygen atoms, respectively. The $E_{av}$ of these systems is very negative, which again reflects the structural stability of the Bi@Cu_2_O(100) on hollow site, in agreement with the tend in $E_{c}$ which is 0.08 eV favorable than that of the pristine Cu_2_O(100) surface. Meanwhile, the distances between the two Bi atoms are respectively 5.27 and 4.798 Å in the case of complete relaxation, which are much larger than the atomic bond length in the Bi clusters [43]. Therefore, the Bi@Cu_2_O(100) on hollow site is more stable than the parent Cu_2_O(100) surface and Bi atoms tend to individual dispersion instead of aggregation on the Cu_2_O(100) surface.

### 3.3. Electronic Structure

**Figure 4:**
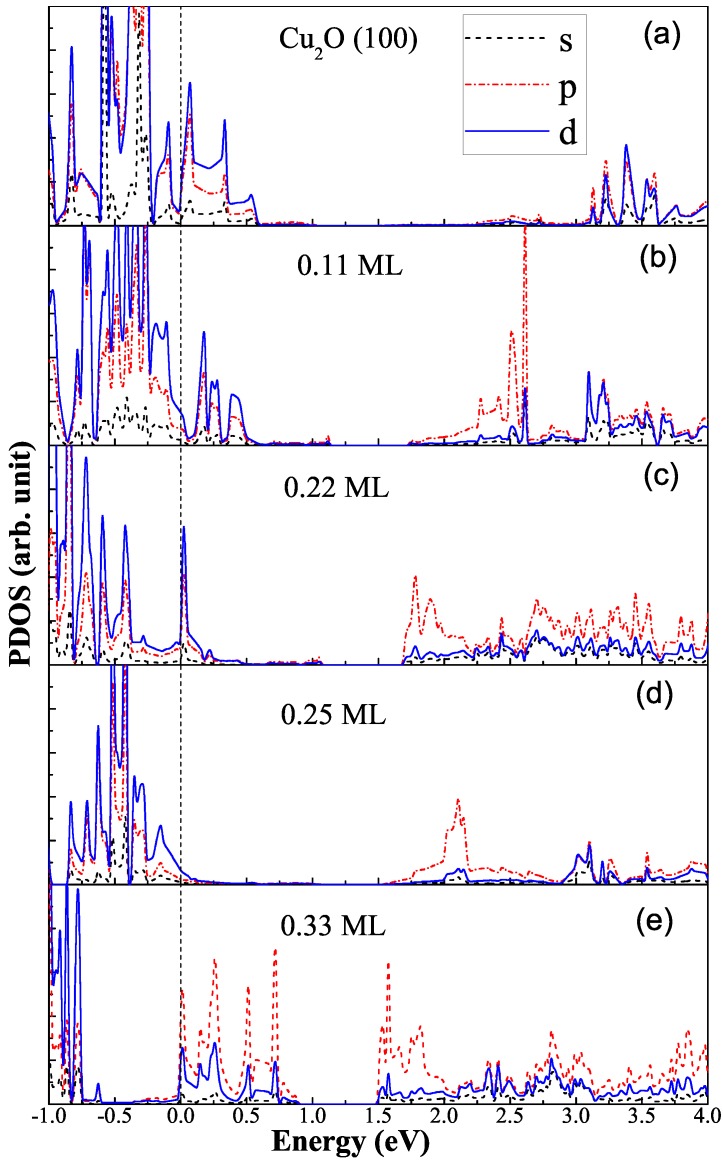
The PDOS of clean Cu_2_O(100) surface and Bi adsorbed Cu_2_O(100) surface. The vertical dashed lines at energy zero indicate the Fermi levels.

**Figure 5:**
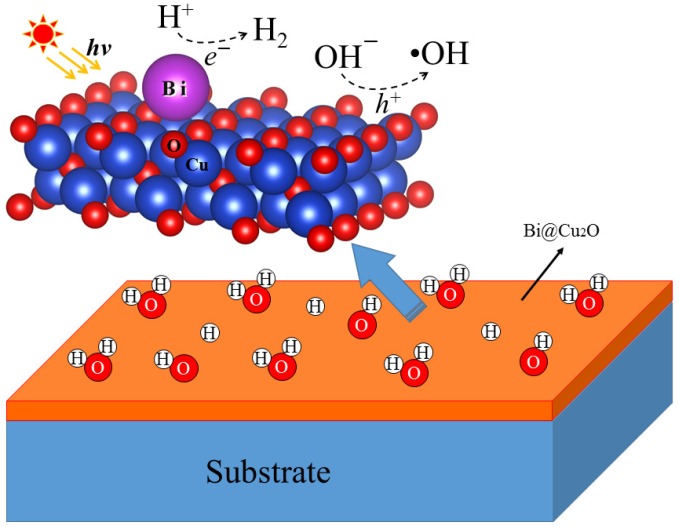
The photocatalytic oxidation and reduction on the Cu_2_O(100) surface.

In order to study the effect of Bi atom adsorption on the electronic structure of Cu_2_O(100) surface, we calculate the PDOS of the clear and Bi-decorated Cu_2_O(100) surface. As a comparison, the PDOS of the clean Cu_2_O(100) surface is shown in Figure 4a. Close to the right side of the Fermi level, there are significant surface states that are not occupied by electrons and they are mainly composed of Cu *d*-orbitals and O *p*-orbitals. This trapping state as a carrier recombination center is not conducive to the photocatalytic reaction [33,34]. Meanwhile, this trapping state grabs electrons from the Cu atoms and oxidizes $Cu^{+}$ to $Cu^{2+}$, which causes Cu_2_O to gradually lose its photocatalytic ability. This is also one of the microscopic mechanisms of photocorrosion of Cu_2_O. Figure 4b shows the PDOS of a single Bi atom adsorbed on the p(3 × 3) surface. For the adsorption of 0.11 monolayer (ML) of Bi atoms, the surface states still exist, but the conduction band edge changes from the *d*-orbitals character of Cu atoms to the *p*-orbitals character of the Bi atom, while the valence band edge keeps the *d*-orbitals character of the Cu atoms. This indicates that the adsorption of Bi atoms successfully change the band edge composition, enabling the band edges to meet the angular momentum selection rules for the light excited transition of electrons. As shown in Figure 4c, when the adsorption concentration is 0.22 monolayer, the surface state is significantly reduced and meanwhile the composition of the band edges also satisfy the transition selection criteria. As is expected, the surface state will be further reduced, when the concentration is increased. As shown in Figure 4d, with a coverage of 0.25 monolayer, the surface state of cuprous oxide is almost completely eliminated, and moreover, the significant *d*-orbitals of Cu atoms at the valence band and the p-orbitals of Bi toms at the conduction band edges meets the light excited electronic transition. Finally, Figure 4e shows the PDOS of Bi absorbed Cu_2_O(100) surface with a coverage of 0.33 monolayer. Since the Bi atomic adsorption concentration is too high, the new impurity states, which are mainly contributed by the *p*-orbital of the Bi atom, appears in the range of 0–0.9 eV. The above analysis of PDOS reveals a trend: with the increase of Bi atom adsorption concentration, the passivation effect of the surface states becomes stronger when the Bi absorption concentration is less than 0.33 ML. To summarize, Bi decoration does not change the bandgap of Cu_2_O(100) surface; however, it can increase the charge carrier density at the band edge, and more importantly it can eliminate the surface states, reducing the carrier recombination and change band edge composition, allowing the transition of *d*-electrons at VBM to *p*-orbitals at CBM under visible light irradiation. It should be pointed out that the modified electronic structure means that the photogenerated electrons will concentrate on the Bi atoms, while the photogenerated holes will concentrate on the Cu atoms. As shown in Figure 5, the Bi atoms become sites of hydrogen evolution reactions to produce hydrogen (H_2_) and the Cu atoms act as sites of oxygen evolution reactions to generate hydroxyl radicals (·OH). Bi decoration successfully separated oxidation reaction and reduction reaction, which reduces the interference of the two reaction processes. This should reduce the recombination rate of photogenerated electron-hole and improve the photocatalytic efficiency. Therefore, the Bi_0.25_@Cu_2_O(100) is the promising photocatalyst.

**Figure 6:**
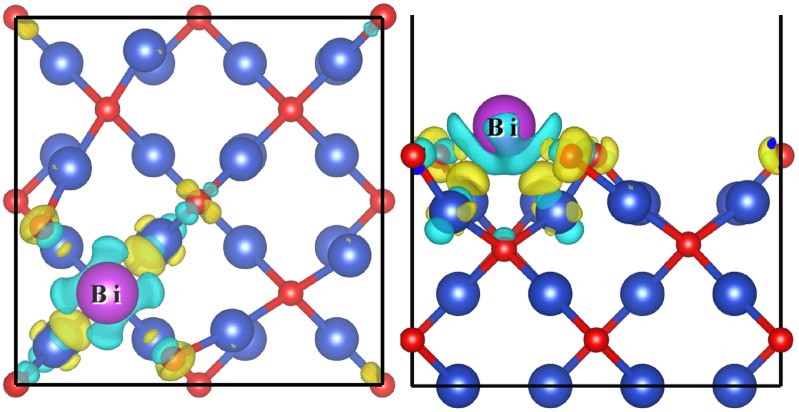
The charge density difference of Bi adsorbed Cu_2_O(100) surface with a coverage of 0.25 ML. Electron accumulation and depletion are represented by yellow and cyan-blue areas, respectively. The isosurface value is set as 0.008 e/Bohr^3^.

The difference of charge density can be obtained with the expression: $\Delta \rho =\rho_{slab+Bi}-\rho_{slab}-\rho_{Bi}$, where $\rho_{slab+Bi}$, $\rho_{slab}$ and $\rho_{Bi}$ are the charge densities of the absorbed system, Cu_2_O substrate and Bi adsorbate, respectively. The charge density difference for Bi_0.25_@Cu_2_O displays in Figure 6. It can be seen that the charge transfer from the Bi atom to the Cu atoms and the O atoms on the surface. The Bader charge analysis shows that the charge of the adsorbed Bi is +1.36e. The charge transfer indicates that Bi atoms form robust chemical adsorption rather than physical adsorption. The strong electron transfer also indicates that the *p*-orbital electrons of Bi atoms hybridize with the *d*-orbital electrons of copper atoms, which passivate successfully the surface trapping states. Meanwhile, the density of states at the conduct band edge increase. Unfortunately, the intensity of electron density weaken at the valence band edge.

### 3.4. Band Edge

**Figure 7:**
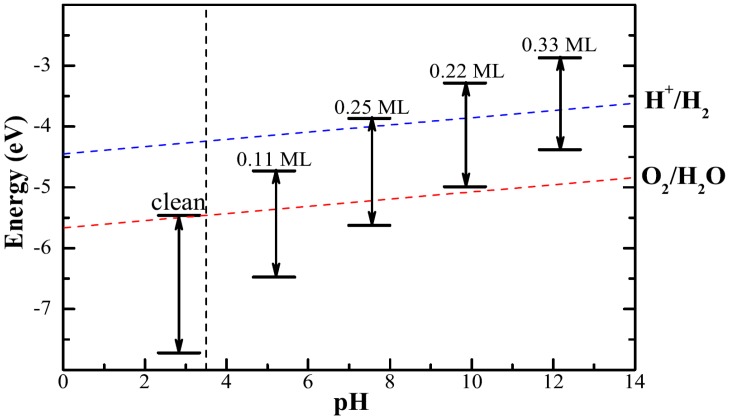
The band edges of clean and adsorbed Cu_2_O(100) surface with respect to the redox potential of water. The blue and red dashed lines represent the reduction potential and the oxidation potential of water as function of pH value, respectively. The black vertical dashed line is at a pH = 3.5.

For hydrogen production via photocatalytic water splitting, the band edges of semiconductor need to be placed appropriately relative to the reaction redox potentials. In order to evaluate the photocatalytic performance, we plot the relative position of the calculated band edge energy levels with respect to water redox potential. In this calculation, the potential in the vacuum region is defined as the reference vacuum level. The voltage for the water splitting reaction is 1.23 V, which is the potential difference between the anodic oxygen evolution reaction and the cathodic hydrogen evolution reaction [44]. The reduction potential of $H^{+}/H_{2}$ is $E_{H^{+}/H_{2}}=-4.44+pH\times 0.059$ eV with reference to the vacuum level [45]. Then the oxidation potential of $O_{2}/H_{2}O$ is $E_{O_{2}/H_{2}O}=E_{H^{+}/H_{2}}-1.23$ eV. The theoretical band edge values of the clean and adsorbed surface are illustrated in Figure 7. For the synthesis of stable Cu_2_O(100) surface, the weak acid conditions (pH = 3.5) [31] required are indicated by vertical dashed line. Cu_2_O(100) surface is not enough to produce hydrogen from photocatalytic water-splitting because of its too low bandedge position. Bi decoration successfully adjusted the position of bandedges. The band edges of Bi_0.25_Cu_2_O straddle the redox potentials of water, making it a desirable band edge position as an excellent photocatalyst for hydrogen generation from water splitting.

### 3.5. Optical Property

The optical absorption spectrum of the material is also a quality factor that reflects the photocatalytic performance. The absorption coefficient is determined by the real and imaginary parts of the frequency dependent complex dielectric function $\varepsilon \left(\omega \right)=\varepsilon_{1}\left(\omega \right)+i\varepsilon_{2}\left(\omega \right)$ according to the following equation [46]:

(3) $$I\left(\omega \right)=\sqrt{2}\omega \sqrt{\sqrt{\varepsilon_{1}^{2}\left(\omega \right)+\varepsilon_{2}^{2}\left(\omega \right)}-\varepsilon_{1}\left(\omega \right)}.$$

**Figure 8:**
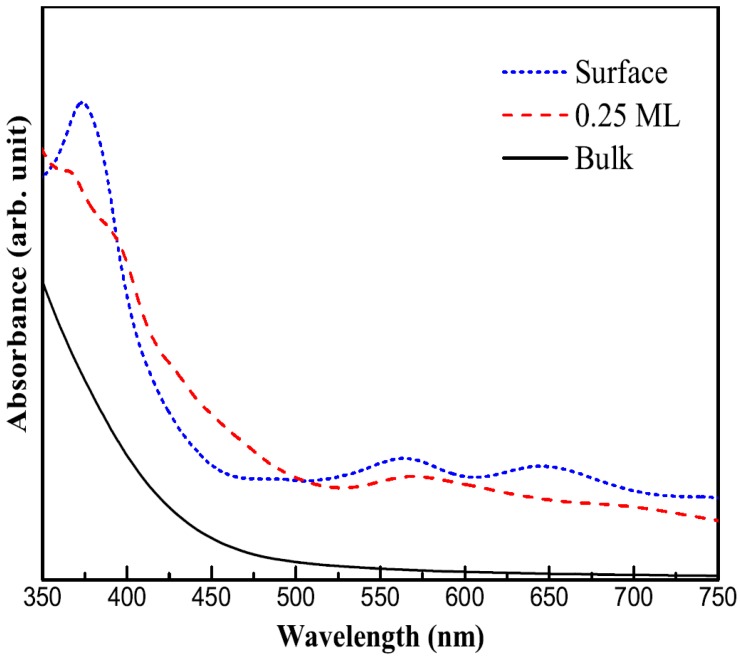
The calculated optical absorption curves of Cu_2_O bulk, clean and Bi adsorbed Cu_2_O(100) surface.

The imaginary part of the dielectric function $\varepsilon_{2}$ is calculated by [47]

(4) $$\varepsilon_{2}\left(\hbar \omega \right)=\frac{2e^{2}\pi }{\Omega \varepsilon_{0}}\sum_{k,v,c}|\langle \psi_{k}^{c}\left|u\cdot r\right|\psi_{k}^{v}{\rangle |}^{2}\delta \left(E_{k}^{c}-E_{k}^{v}-\hbar \omega \right),$$

where Ω, ω, u, $\psi_{k}^{v}$ and $\psi_{k}^{c}$ are the volume, photon frequencies, the vector defining the polarization of the incident electric field, the occupied and unoccupied wave functions at point *k* in reciprocal space, respectively. The real part $\varepsilon_{1}$ can be evaluated from imaginary part $\varepsilon_{2}$ by the Kramer–Kronig relationship [48]:

(5) $$\varepsilon_{1}\left(\omega \right)=1+\frac{2}{\pi }p\int_{0}^{\infty }\frac{\varepsilon_{2}\left(\omega^{{}^{\prime }}\right)\omega^{{}^{\prime }}}{\omega^{{}^{\prime }2}-\omega^{2}}d\omega^{{}^{\prime }},$$

where *p* is the principal value of the integral. The calculated optical properties of all the systems are shown in Figure 8. The optical absorption curves indicate that the adsorption of Bi atoms on the Cu_2_O(100) surface has little effect on the optical absorption. Around 400 nm, which corresponds to visible light, there is an absorption-curve platform suggesting that the system still maintains a positive visible light response. The residual impurity states in the band gap cause some small absorption peaks above 500 nm and cause the absorption limit to move to the infrared region. The introduction of Bi atoms does not damage the good optical properties of the cuprous oxide, and the adsorption of Bi atoms is likely to be a kind of reliable means of modification for cuprous oxide.

## 4. Conclusions

The first-principles calculations based on hybrid density functional theory are performed to investigate the electronic structure of pure and Bi atoms deposited Cu_2_O(100) surface. The calculation results reveal the presence of surface trapping states on the clean Cu_2_O(100) surface. The surface trapping states promote the recombination of the excited electron- hole and inhibit the photocatalytic efficiency in practical application. In order to improve the photocatalytic activity of Cu_2_O, we propose a strategy to passivate trapping states by depositing Bi atoms on Cu_2_O(100) surface. The adsorption of Bi atom on the hollow site among four surface oxygen atoms is most stable. For the different surface coverage, the absolute adsorption energy increases as the surface coverage increases. In the case of low surface coverage, the even-parity symmetry of the band edges has changed to meet the angular momentum selection rules for the light excited transition of electrons; But the surface trapping states are still maintained, indicating that the passivation effect is not sufficient. When the adsorption concentration increases to 0.25 ML, not only the surface trapping states are passivated, but also the band edges become satisfactory. Moreover, our scheme helps to reduce the recombination rate of photogenerated electron-hole and improve photocatalytic efficiency. We attempted to continue increasing the Bi atomic adsorption concentration and found a new impurity state from Bi atoms at a surface coverage of 0.33 ML. In addition, the band edges straddle the reaction redox potentials with a surface coverage of 0.25 ML. Finally, the analysis of optical absorption spectrum shows that these systems still retains their response to visible light. Therefore, the surface coverage of 0.25 ML is an ideal adsorption concentration in theoretical calculations for the highly efficiency of visible light photocatalysis. We believe that our findings will provide a new possibility for photocatalytic experiments.

## Figures and Tables

**Table 1:** Adsorption energy and surface cohesion energy for bismuth on the Cu_2_O(100) surface.

Systems	θ	Adsorption Site	$E_{\mathrm{av}}$	$E_{c}$
	(ML)		(eV/Å^2^)	(ev/Å^2^)
Cu_2_O(100)	0	-	-	−2.01
p (2 × 2)	0.25	O-top	−0.04	−2.05
p (2 × 2)	0.25	O-Bridge	−0.07	−2.08
p (2 × 2)	0.25	Cu_1_-top	−0.05	−2.06
p (2 × 2)	0.25	Cu_2_-top	−0.05	−2.06
p (2 × 2)	0.25	Hollow	−0.08	−2.09
p (3 × 3)	0.11	Hollow	−0.04	−2.05
p (3 × 3)	0.22	Hollow	−0.08	−2.09
p (3 × 3)	0.33	Hollow	−0.09	−2.10
